# A dataset on the sensory and affective perception of Bordeaux and Rioja red wines collected from French and Spanish consumers at home and international wine students in the lab

**DOI:** 10.1016/j.dib.2022.108873

**Published:** 2022-12-31

**Authors:** Michel Visalli, Magalie Dubois, Pascal Schlich, François Ric, Jean-Marie Cardebat, Nikolaos Georgantzis

**Affiliations:** a Centre des Sciences du Goût et de l'Alimentation, AgroSup Dijon, CNRS, INRAE^1^, Université Bourgogne Franche-Comté, Dijon F-21000, France; b INRAE, PROBE research infrastructure, ChemoSens facility, Dijon F-21000, France; c Burgundy School of Business, CEREN, EA 7477, Université Bourgogne Franche-Comté, 29 rue Sambin, BP 50608, Dijon Cedex 21006, France; d Avenue Leon Duguit - Bâtiment H, Université de Bordeaux, BSE (UMR CNRS 6060), Pessac 33608, France; e INSEEC School of Business and Economics, H19, quai de Bacalan, Bordeaux 33000, France; f Faculté de Psychologie et Laboratoire de Psychologie (EA4139), University of Bordeaux, Bordeaux 33000, France

**Keywords:** Sensory analysis, Liking, Free-comment, Temporal method, Willingness to pay, Cross-cultural, Impact of information, Incentive-compatible experiment

## Abstract

This article describes a dataset providing temporal sensory descriptions and affective answers for red wines: two Bordeaux and two Riojas. The wines were tasted at home by French (FR, n=106) and Spanish (SP, n=98) consumers and in the lab by wine students (WC, n=47). Standardized information was displayed on the samples (country and region of origin, name, producer, vintage, alcohol content). The FR and SP panels were split into three groups, the first having no rating information, the second having expert rating information (based on Wine Advocate ratings), and the third having consumer rating information (based on online Vivino reviews). The participants first rated their expected liking for the four wines. Then, for each wine sample, they had (in order) to taste the sample while being video recorded, rate their liking, temporally describe the sequence of sensations they perceived using Free-Comment Attack-Evolution-Finish, answer several questions about familiarity and quality perception, and declare their willingness to pay (reserve price). Then, they had to rank the four wines according to their quality. General questions about wine involvement, subjective wine knowledge, valuation behaviour, purchasing, and consumption patterns were asked. Finally, an auction was resolved: participants declaring a reserve price greater than the drawn price won a bottle. The data were used to assess the influence of culture and expertise on temporal sensory evaluations in an article entitled “Using Free-Comment to investigate expertise and cultural differences in wine sensory description”. The data can be reused by researchers interested in studying the impact of external information on preferences and choices or investigating the sensory drivers of liking.

**Specifications Table**

**Table:** 

Subject	Food science
Specific subject area	Wines
Type of data	Tables Questionnaire Figures
How the data were acquired	Sensory data were acquired by recruiting two panels of consumers (98 Spanish, 106 French) at home through Qualtrics and one panel of wine students (47 international students) using a web application.
Data format	Tables in raw format (XLSX file)
Description of data collection	Three panels tasted two Bordeaux and two Rioja red wines. Standardized information was displayed on the samples (origin, designation, producer, vintage, alcohol content). The French and Spanish panels were split into three groups, the first having no rating information, the second and third having additional external information, respectively expert rating and consumer rating for the evaluated sample. The participants first rated their expected liking for the four wines on a 7-point scale. Then, for each wine sample, they had (in order) to taste the sample while being video recorded; rate their liking on a 7-point scale; temporally describe the sensations they perceived using Free-Comment Attack-Evolution-Finish; answer several questions (about their familiarity, perception of quality, perception of others’ liking); and declare their willingness to pay (reserve price). Then, they had to rank the four wines according to their quality. General questions about their behaviour towards wines were asked (interest, choice, knowledge, purchases, consumption).
Data source location	• City/Town/Region: Dijon (students), everywhere in France or Spain (consumers) • Country: France, Spain
Data accessibility	Repository name: Mendeley data Data identification number: 10.17632/f9wtj7s9b8.1 Direct URL to data: https://data.mendeley.com/datasets/f9wtj7s9b8/1
Related research article	M. Visalli, M. Dubois, P. Schlich, F. Ric, J.M. Cardebat, N. Georgantzis. Using Free-Comment to investigate expertise and cultural differences in wine sensory description. Food Quality and Preference.

## Value of the Data

These data are useful because they provide information about wines’ perceived quality, stated and revealed preferences collected from consumers and experts from different countries under different information conditions.

Researchers or product developers can reuse these data to test the impact of external information (peer and expert ratings) on sensory perception, preferences or willingness to pay for wines. They can also study wheter this impact varies according to the expertise and culture of the participants.

They can compare the information obtained with a sensory evaluation with that available in online wine reviews. They can also benefit from these data to investigate the drivers of liking for red wines.

## 1 Objective

This dataset has been generated in order to compare the expectations, temporal sensory perception, willingness to pay, and preferences for two Bordeaux and two Rioja red wines tasted in two settings (at home and in the lab) by three panels varying in culture and expertise and having different informations about the wines. The research article associated with this data paper only reports results on temporal sensory perception.

## 2 Data Description

The dataset is provided as an Excel file (.xlsx) including five sheets:

***Participants*** provides information about the participants collected during the screening and in the questionnaire.

“Panel” is the panel to which the participant has been assigned (WC: wine connoisseurs, FR: French consumers, SP: Spanish consumers).

“Language” is the language of the participant. “Participant” is the unique anonymized identifier of the participant.

“Age” is the age range of the participant (18-29, 30-40, 41-50, 51-60, 61-70, 71 and older).

“Gender” is the gender of the participant (Male, Female, or Other).

“PCS” is the socioprofessional category of the participant (Employed, Unemployed, Student, Retired, Other).

”Diploma” is the higher level of diploma obtained by the participant (None, High school, Vocational school, Bachelor's degree, Master's degree, PhD, Other).

“Income” is the income-related quality of life estimated by the participant (Living comfortably on present income, Coping on present income, Finding it difficult on present income, Finding it very difficult on present income, I prefer not to answer).

“Group” is the group of the participant (No rating information, Consumer rating information, Expert ratings information).

“QuestionnaireBeginDate” is the date (YYYY-MM:DD hh:mm:ss) when the participant started the study (first connection).

“QuestionnaireEndDate” is the date (YYYY-MM:DD hh:mm:ss) when the participant ended the study.

“PCI1”, “PCI2” and “PCI3” are related to product category involvement [1].

“PC1” is the answer to the question “Wine interests me a lot” rated on a 5-point Likert scale (1: strongly disagree, 2: disagree, 3: neither agree nor disagree, 4: agree, 5: strongly agree).

“PCI2” is the answer to the question “I often discuss wine with other people” rated on a 5-point Likert scale (same values as PCI1).

“PCI3” is the answer to the question “It gives me pleasure to shop for wine” rated on a 5-point Likert scale (same values as PCI1).

“SWK1”, “SWK2”, “SWK3” and “SWK4” are related to subjective wine knowledge [2].

“SWK1” is the answer to the question “I feel confident in my ability to choose wine” [3] rated on a 5-point Likert scale (same values as PCI1).

“SWK2” is the answer to the question “I know more about wine than many other people” rated on a 5-point Likert scale (same values as PCI1).

“SWK3” is the answer to the question “I would describe myself as being very knowledgeable about wine” rated on a 5-point Likert scale (same values as PCI1).

“SWK4” is the answer to the question “Did you already follow a wine education course?” (1: no, 2: yes, without certification, 3: yes, with certification).

“CP1” and “CP2” are related to consumption patterns [4].

“CP1” is the answer to the question “How often do you consume wine at home?” (1: daily, 2, at least once a week, 3: at least once a month, 4: less than once a month, 5: never).

“CP2” is the answer to the question “How often do you consume wine outside of home (restaurant, bar, club, etc.)?” (same values as CP1).

“AC1” is the answer to the question “If you read this sentence correctly, please answer ‘strongly disagree’” [5] rated on a 5-point Likert scale (same values as PCI1, expected answer=1).

“VB1”, “VB2”, “VB3” and “VB4” are related to valuation behaviour [6].

“VB1” is the answer to the question “I use wine apps to help me decide which wine to buy” (1: never, 2: once in a while, 3: often, 4: always).

“VB2” is the answer to the question “I use wine professional expert ratings (wine reviews, point scores, medals, and awards) to help me decide which wine to buy” (same values as VB1).

“VB3” is the answer to the question “I often seek advice from other people before purchasing a wine” rated on a 5-point Likert scale (same values as PCI1).

“VB4_1” to “VB4_8” are the answers to the question “Whose advice do you trust most when selecting a wine?” (VB4_1: Friends, VB4_2: Family members, VB4_3: Colleagues, VB4_4: Sommelier, VB4_ 5: Professional Wine Expert, VB4_6: Wine blogger or influencer, VB4_7: Wine Guide or Magazine, VB4_8: Only my own). Answers are 1 if the option was checked and 0 otherwise.

“PP1”, “PP2”, “PP3”, and “PP4” are related to purchasing patterns [7].

“PP1_1” to “PP1_7” are the answers to the question “Where do you buy your wine?” (PP1_1: I do not buy wine, PP1_2: Supermarket, PP1_3: Wine store, PP1_4: Online, PP1_5: Directly from the winemaker, PP1_6: Restaurants and bars, PP1_7: Other). Answers are 1 if the option was checked and 0 otherwise.

“PP2” is the answer to the question “For a 75-cl bottle of red wine, you spend on average - for informal drinking” (1: I do not buy wine, 2: less than 5€, 3: 5-10€, 4: 11-20€, 5: 21-30€, 6:30€ and more).

“PP3” is the answer to the question “For a 75-cl bottle of red wine, you spend on average - for a formal occasion or a gift” (same values as PP2).

“PP4” is the answer to the question “How much does your household spend on wine monthly?” (1: 0€, 2: 50€ or less, 3: 51-100€, 4: 101-150€, 5: 151€ or more).

***Qualities*** provides information about the subjective qualities evaluated for each wine by the participants.

“Panel” and “Participant” are the same as in the “Participants” tab.

“Wine” is the code of the evaluated wine.

“EL1” is the answer to the question “How much do you think to like this wine?” [8], rated on a 7-point hedonic scale (1: dislike extremely, 2: dislike moderately, 3: dislike slightly, 4: neither like nor dislike, 5: like slightly, 6: like moderately, 7: like extremely).

“SL1” is the answer to the question “How much did you like this wine?” [8], rated on a 7-point hedonic scale (same values ac EL1).

“QE1” is the answer to the question “I think this wine is high quality”, rated on a 5-point Likert scale (same values as PCI1).

“QE2” is the answer to the question “Most people would like this wine”, rated on a 5-point Likert scale (same values as PCI1).

“F1” is the answer to the question “This wine profile is familiar to me”, rated on a 5-point Likert scale (same values as PCI1).

“RQ1” is the answer to the question “Rank the 4 wines by clicking on ‘click to choose a wine’ and then giving each wine a rank. #1=most qualitative wine, #4=least qualitative wine”.

“BDM1” is the answer (reserve price) to the question “What is the maximum price (in €uros) you are willing to pay for a 75cl bottle of the wine you just tasted?”) [9].

***TemporalPerception*** provides information about temporal sensory perception evaluated for each wine by the participants.

“Panel” and “Participant” are the same as in the “Participants” tab.

“Wine” is the code of the evaluated wine.

“Period” is the code of the period defined in the question (AEF1: “At first, I perceived this wine”, AEF2: “Then, after a few moments, I perceived it”, AEF3: “At the end of the tasting, I perceived it”).

“Description” is the Free-Comment description of “Product” at “Period” depending on the period, as entered by “Participant” (in English, French or Spanish).

“Keywords” contains the lemmas related to sensory attributes (canonical form, masculine, singular), separated by commas, translated into English (if required).

***AuctionResolution*** provides information about the results of the auction.

“Panel” and “Participant” are the same as in the “Participants” tab.

“RandomProduct” is the code of the randomly drawn wine (between W1, W2 or W4, W3 being not available at the end of the study).

“RandomPrice” is the price randomly drawn in the distribution of the prices of red wines of Bordeaux and Rioja (extracted from Vivino).

“Result” was the result of the auction (win if RandomPrice **≥** reserve price for RandomProduct, lose otherwise).

***Questionnaire 1*** includes commented screenshots of the online questionnaire used to collect data, translated from French and Spanish to English.

Table 1 provides objective information about wines.

**Table 1:** Objective information about wines. AOC: Appellation d'Origine Controllée (protected designation of origin). DOP: Denominación de Origen Protegida (protected designation of origin).

Code	Appellation	Winery	Variety	Vintage	Alcohol Content	Price in euros
W1	AOC Bordeaux Supérieur	Chateau Féret Lambert	Merlot 90%, Cabernet 10%	2018	14.5	15.5
W2	AOC Pessac Léognan	La Louvière	Merlot 40%, Cabernet Sauvignon 60%	2018	13.5	15.6
W3	DOP Rioja	Bhilar	Tempranillo 85%, Grenache 10%, Viura 5%	2018	13.5	16
W4	DOP Rioja	Miguel Merino	Tempranillo 100%	2018	14.0	14.5

Table 2 provides subjective information about wines collected on the Robert Parker Wine Advocate [10] and Vivino [11] websites in May 2022.

**Table 2:** Subjective information about wines.

Code	Parker score	Vivino score	Parker description	Vivino description
W1	85/100	3.76/5 (383 notes)	“Deep garnet-purple colored, the 2018 Feret-Lambert leaps from the glass with crème de cassis, boysenberries and black raspberries followed by plum pudding and cloves nuances. Full-bodied, it coats the mouth with dried berries and exotic spice flavors, framed by chewy tannins and just enough freshness, finishing earthy”. (Lisa Perrotti-Brown, 23rd Apr 2019)	Light/Strong: 8.5/10; Supple/Tannic: 6.5/10; Dry/Liquorous:0.5/10; Sweet/Acid: 7/10; Prune, blackberry, black fruit (79%); Oak, vanilla, tobacco (46%); Earthy, leather, smokey (41%); Cherry, raspberry, red berries (32%); Pepper, licorice, anise (14%)

W2	94/100	4.07/5 (84 notes)	“Medium to deep garnet-purple in color, the 2018 la Louviere leaps from the glass with notions of redcurrant jelly, fresh blackberries and warm black plums, plus nuances of dried mint, cedar chest and ground cloves. The medium-bodied palate is refreshing and savory in the mouth, featuring a light touch of finely grained tannins and bold freshness, finishing with a compelling red berry lift. It's an elegant, lively expression of this vintage and one that really works! “(Lisa Perrotti-Brown, 31st Mar 2021)	Light/Strong: 8/10; Supple/Tannic: 7.5/10; Dry/Liquorous:0.5/10; Sweet/Acid: 8.5/10; Oak, vanilla, tobacco (37%); Somey, leather, cocoa (21%); Blackberry, black fruit, blackcurrant (19%); Cherry, strawberry, sour cherry (14%); Licorice, pepper, anise (13%)

W3	88/100	3.21/5 (16 notes)	“It has a strong note of cider on the nose, with a volatile touch and a nutty touch and very low alcohol.” (Luis Gutiérrez, 28th Jun 2019)	Light/Strong: 4.5/10; Supple/Tannic: 6/10; Dry/Liquorous:3.5/10; Sweet/Acid: 6.5/10; Blackcurrant (50%)

W4	90/100	4.10/5 (135 notes)	“The wine is juicy, very drinkable but serious, with fantastic balance and very clean aromas and flavors. It's quite fruit driven but has the complexity of a more serious wine produced with attention to detail. It already has Rioja character.” (Luis Gutiérrez, 30th Oct 2020)	Light/Strong: 7/10; Supple/Tannic: 6.5/10; Dry/Liquorous:2/10; Sweet/Acid: 6.5/10; Vanilla, oak, chocolate (41%); Prune, black cherry, black fruit (27%); Cocoa, leather, earthy (21%); Cherry, strawberry (14%); Pepper, licorice, anise (7%)

Table 3 describes the individual characteristics of the participants in the three panels by group.

**Table 3:** Participant characteristics.

	WC	FR no rating	FR consumer rating	FR expert rating	SP no rating	SP consumer rating	SP expert rating
Age 18-29	57%	7%	11%	13%	6%	10%	6%
Age 30-40	21%	17%	18%	15%	37%	30%	27%
Age 41-50	15%	21%	13%	26%	23%	30%	27%
Age 51-60	4%	17%	16%	13%	23%	13%	18%
Age 61-70	2%	31%	32%	21%	11%	17%	18%
Age 70+	-	7%	11%	10%	-	-	3%
Female	62%	59%	48%	46%	46%	60%	52%
Male	36%	41%	53%	52%	54%	40%	48%
Other	2%	-	-	3%	-	-	-
Employed	11%	55%	55%	59%	77%	73%	79%
Other	-	14%	-	3%	3%	3%	3%
Retired	-	28%	37%	26%	9%	3%	8%
Student	89%	3%	5%	5%	-	-	6%
Unemployed	-	-	3%	8%	11%	20%	3%
PhD	4%	-	-	8%	3%	7%	6%
High school	-	34%	24%	23%	6%	13%	12%
Bachelor's degree	51%	17%	17%	13%	49%	33%	48%
Master degree	43%	17%	24%	21%	20%	30%	21%
None	-	-	-	5%	-	-	-
Other	2%	-	5%	-	-	3%	-
Vocational school	-	31%	32%	28%	23%	13%	12%
Living comfortably	34%	10%	24%	23%	45%	40%	45%
Finding it difficult	9%	21%	13%	15%	12%	3%	12%
No answer	23%	-	3%	3%	-	-	-
Coping on income	34%	69%	61%	59%	42%	57%	42%

**Fig. 1:**
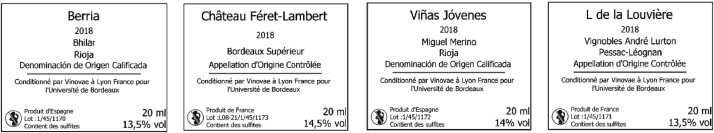
Standardized labels of the samples.

Fig. 1 shows the standardized labels displayed on the wine samples.

**Fig. 2:**
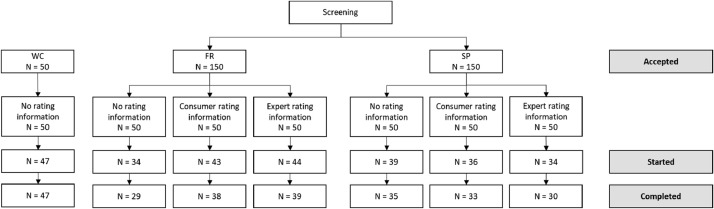
Participant flowchart.

Fig. 2 is the flowchart of the participants (recruited, participated, completed).

## 3 Experimental Design, Materials and Methods

### 3.1 Samples

The four products (W1, W2, W3, and W4) were nonorganic red wines produced in France or in Spain.

The 750 ml bottles were purchased from an online store.

The wines chosen came from two regions to study the cultural impact and have noncongruent Vivino and Parker scores to study the impact of information (expert or consumer ratings from reviews). The year of production was the same, and the prices were very close.

The reviews in Wine Advocate were conducted by highly experienced tasters, specially trained to understand and recognize wine quality in a glass. They used 100-point quality scales to evaluate the wines. The Vivino taste profiles of wines were based on user reviews. The values reported in Table 2 were measured on the bipolar scales (“Light/strong”, “Supple/Tannic”, “Dry/Liquorous”, “Sweet/Acid”) displayed on the website and converted between 0 and 10. The percentages associated with the flavours correspond to the percentages of consumers who cited the flavour according to Vivino's counts.

Vinovae [12] used a patented process to repackage the bottles in 20 ml polyethylene terephthalate (PET) screw bottles in an inert atmosphere devoid of oxygen. The process was designed to avoid the risk of oxygenation and ensure the preservation of the organoleptic qualities of the wines. The screw bottles were labelled with original information (origin, designation, producer, vintage, alcohol content) and displayed in a standardized way. The samples were sent to the consumers’ houses by postal mail.

### 3.2 Participants

“Wine connoisseur” panel (WC): Fifty students of the School of Wine and Spirits Business of the Burgundy School of Business in Dijon were recruited through a mailing. They were natives from different countries and selected based on their knowledge about wines (they were at least holders of WSET certificate (Wine and Spirits Education Trust) Level 2 Award in Wines [13]).

French (FR) and Spanish (SP) consumer panels: A total of 150 French consumers and 150 Spanish consumers were recruited from a panel recruitment agency database through online questionnaires (Qualtrics). The selection criteria included (i) being available to participate in a 20-minute online study involving the at-home tasting of four wines; (ii) having consumed red wine at home within the past month; (iii) possessing at least one wine glass at home; (iv) owning a computer with a webcam and a good internet connection (Chrome, Firefox or Edge browser); (v) agreeing to be video recorded during the tasting; and (vi) agreeing to provide a postal address for the shipment of samples. Quotas on iindividual characteristics (age, gender, employment status, education level, family income) were also established to balance the two consumers panels. The appropriate sample size was determined based on the literature [14].

All participants (WC, FR, SP) signed an informed consent form. They were informed that they would receive compensation worth 20 euros (that may include a bottle of wine depending on the resolution of an auction), and they could refuse to participate or stop participating in the study at any time without providing a reason; however, in that case, they would not receive any compensation. They were also informed that the study was an academic research project without any commercial interests and that the information collected would be used exclusively for research purposes.

Consumers in FR and SP panels were both divided into three groups of 50: the first (control group) had no other information than the label (“no rating information”), the second received information about expert ratings (“expert rating information”), the third received information about consumer ratings (“consumer rating information”).

Forty-seven (94%) students finally completed the study in the WC panel, 106 consumers (70%) in the FR panel, and 98 (65%) in the SP panel.

### 3.3 Data collection

The consumers received an email containing an individualized URL to invite them to connect to the TimeSens version 2 web application [15] using a web browser (Chrome, Firefox, or Edge were recommended to ensure maximum compatibility with the web app) on their computer. The FR and SP panels completed the experiment at home, and the WC panel completed the experiment in the sensory lab of the Burgundy School of Business (Dijon, France, 32 available individual boxes) during three sessions.

The experimental procedure followed the steps of the questionnaire described below.

Screen 1: reading and acceptance of the conditions of the study.

Screen 2: reminder that four wines had to be evaluated and that the tasting part would be video recorded.

Screen 3: rating of expected liking of the four wines using a 7-point hedonic scale (question EL1).

Screen 4: instructions to prepare by having a glass of water and an empty wine glass available for the tasting.

Screen 5: instructions for webcam calibration included facing the webcam; having the face and forefront visible (no glasses); adapting the light to be homogeneous; having the face occupy 25 to 30% of the screen; avoiding white clothing, direct lighting, a dark environment, and anything that masks the face; and turning off the phone during the study.

Screen 6: instruction checklist displayed on screen 5.

Screen 7: displaying the video flux of the webcam to adjust the calibration of the webcam.

Screen 8: instructions for water tasting (warm-up) included pouring some water in the wine glass; looking at the water, swirling and sniffing it; and taking a small mouthful and at the same time clicking on the button to start the video recording.

Screen 9: displaying the video flux from the webcam during the water tasting (ten seconds).

Screen 10: instructions for preparing a new sample included emptying the wine glass; pouring the appropriate wine sample in the glass (the order of presentation of the samples was balanced over participants based on a William's Latin square); looking at the wine, swirling and sniffing it; and taking a small mouthful and at the same time clicking on the button to start the video recording. The label corresponding to the wine sample they had to taste was displayed on the screen (see Fig. 1). The participants in the “expert rating information” group received the following supplementary information to the right of the sample label: “This wine was scored x/100 by the Wine Advocate – Robert Parker”. The participants in the “consumer rating information” group also received supplementary information: “This wine was rated x/5 by consumers – Vivino website”.

Screen 11: displaying the video flux from the webcam during the wine tasting (ten seconds). This was the only moment when a wine sample was tasted.

Screen 12: rating of liking on a 7-point hedonic scale (question SL1).

Screen 13: explanation of the wine description task, a Free-Comment Attack-Evolution-Finish (FC-AEF) [16]. The participants were informed that they had to retrospectively describe the sensations they perceived in mouth during the tasting in chronological order. Three periods were defined to summarize the tasting: “at first”, “after a few moments” and “at the end of the tasting”. For each period, they had to describe their sensations (tastes, aromas) using their own words. The same words could be used in different periods. A fictive example with chocolate was shown to help the participants understand the task.

Screen 14: FC-AEF task (questions AEF1, AEF2 and AEF3), as explained on screen 13.

Screen 15: rating of question F1 (“The wine I just tasted is similar to the wines I normally select”).

Screen 16: rating of questions QE1 (“I think this wine is high quality”) and QE2 (“Most people would like this wine”).

Screen 17: explanation of the Becker–DeGroot–Marschak (BDM) method [17]. The participants were instructed they would have to propose a price corresponding to the maximum price they would pay for a 75-cl bottle of the tasted wine. They were informed that they could indicate 0 if they did not like the product and did not wish to buy it. They were told that an auction would happen only for one of the four wines randomly drawn at the end of the survey. For this wine, if the price drawn was higher than the indicated price, the participant would lose the auction and would not receive the wine. If the price drawn was lower than the indicated price, the participant would win the auction and receive the bottle in the following few days.

Screen 18: scoring of their maximum willingness to pay for a 75-cl bottle (question BDM1), as explained on screen 17.

Screen 19: instructions for glass rinsing.

Screen 20 to 42: the procedure described on screens 10 to 19 was repeated for the three other wines (explanation screens 13 and 17 were displayed once).

Screen 43: ranking of the four wines (no ex-aequo allowed, question RQ1).

Screen 44: rating of questions PC1 (“Wine interests me a lot”), PCI2 (“I often discuss wine with other people”) and PCI3 (“It gives me pleasure to shop for wine”).

Screen 45: rating of questions SWK1 (“I feel confident in my ability to choose wine”), SWK2 (“I know more about wine than many other people”), SWK3 (“I would describe myself as being very knowledgeable about wine”) and SWK4 (“Have you already taken a wine education course?”).

Screen 46: answering questions VB1 (“I use wine apps to help me decide which wine to buy”) and VB2 (“I use wine professional expert ratings (wine reviews, point scores, medals and awards) to help me decide which wine to buy”).

Screen 47: rating of question VB3 (“I often seek advice from other people before purchasing a wine”).

Screen 48: answering question VB4 (“Whose advice do you trust most when selecting a wine?”, multiple answers authorized).

Screen 49: answering question PP1 (“Where do you buy your wine?”, multiple answers authorized).

Screen 50: answering questions PP2 (“For a 75cl bottle of red wine you spend on average - for informal drinking”), PP3 (“For a 75cl bottle of red wine you spend on average - for a formal occasion or for a gift”) and PP4 (“How much does your household spend on wine monthly?”).

Screen 51: answering questions CP1 (“How often do you consume wine at home?”); CP2 (“How often do you consume wine outside from home (restaurant, bar, club, etc.)?”) and AC1 (“If you read this sentence correctly, please answer ‘strongly disagree’”).

Screen 52: drawing of the wine and the random price and resolution of the auction.

Screen 53: study debriefing.

Screen 54: end screen.

## Ethics Statements

Each participant was informed of the conditions for participating and validated an informed consent form. The research was carried out in accordance with the Declaration of Helsinki and received approval from the Burgundy School of Business Research Ethics Committee (reference of the application: CERBSB2022-9).

## CRediT authorship contribution statement

**Michel Visalli:** Conceptualization, Data curation, Formal analysis, Methodology, Software, Validation, Visualization, Writing – original draft. **Magalie Dubois:** Conceptualization, Data curation, Funding acquisition, Investigation, Methodology, Project administration, Resources, Supervision, Validation, Writing – review & editing. **Pascal Schlich:** Writing – review & editing. **François Ric:** Writing – review & editing. **Jean-Marie Cardebat:** Funding acquisition, Writing – review & editing. **Nikolaos Georgantzis:** Writing – review & editing.

## Declaration of Competing Interest

The authors declare that they have no known competing financial interests or personal relationships that could have appeared to influence the work reported in this paper.

## Data Availability

A dataset on the sensory and affective perception of Bordeaux and Rioja red wines collected from French and Spanish consumers at home and international wine students in the lab. (Original data) (Mendeley Data).

## Appendix Supplementary materials

Image, application 1

Image, application 2
