# Supplementary material for: A dataset on the sensory and affective perception of Bordeaux and Rioja red wines collected from French and Spanish consumers at home and international wine students in the lab
Source: Data Brief. 2022 Dec 31;46:108873. doi: 10.1016/j.dib.2022.108873 (PMC9850030; doi:10.1016/j.dib.2022.108873)
Supplement: Supplementary file 2 [file mmc2.pdf]

Project Title

Servitization and digitization of wine tasting and quality evaluation

Researcher in charge

Magalie Dubois, magalie.dubois@u-bordeaux.fr

Principal Investigator

Magalie Dubois

Description

This study contributes to the research activities of the VinCi project and is targeted at anyone interested in wine consumption. The results of the study will contribute to our understanding of determinants of the wine selection in decision-making processes. More details about the objectives and the outcomes of the VinCi project are available at http://vincisudoe.eu/

You freely and voluntarily accept to participate in the wine study organised by the University of Bordeaux. Consequently, you will participate in a session during which you will taste wines and answer different questions. The 4 phases of the

I DO NOT ACCEPT | I HAVE READ AND I ACCEPT

you will participate in a session during which you will taste wines and answer different questions. The 4 phases of the tasting will be filmed. At the end of the study, you will receive a compensation worth 20 euros.

During this session, you will have the opportunity to buy a bottle of one of the wines presented to you at a price equal to c lower than the price you have declared as the maximum price you are willing to pay for this product. You have noted that you are not obliged to buy a product, but once you offer a price for a product, you have noted that you will be obliged to pay it with a share of the compensation provided.

You undertake to follow scrupulously all the instructions given to you during the session and, if necessary, to pay for the product at the price fixed according to the system explained to you and according to the maximum price you will have given, with a share of the compensation provided.

If you so wish, you may interrupt your participation in this session at any time without giving any justification. In this case, you will not receive any compensation.

Funding for this research

This research is part of the VinCi project, funded by the EU Commission through H2020 funds. SUDOE- SOE3/P2/F0917

I DO NOT ACCEPT | I HAVE READ AND I ACCEPT

Voluntary Participation

Your participation in the research is completely voluntary. The activity will take around 20 minutes.

Data policy

All personal data will be handled according to national and European data protection rules including the General Data Protection Regulation (GDPR) 2016/679. Data processing will be limited to the purposes of research performed in the project.

VinCi will not collect or process any sensitive personal data that reveal racial or ethnic origin, political opinions, religious or philosophical beliefs, trade-union memberships, genetic data, biometric data, data concerning health and data relating to sexual orientation or activity.

Confidentiality

You will be assigned a code number that will be used to match your responses to the computer. All information will be recorded anonymously. No one will be able to divulge your name or identify your answers. All information will be held in the strictest of confidence. Results from the research will be reported as aggregate data to the funding agency and will be used for publishing in international scientific peer-reviewed journals. At the end of the research period, data will be retained by the researcher and will not be sold to a third party but could be shared for further research.

I DO NOT ACCEPT | I HAVE READ AND I ACCEPT

used for publishing in international scientific peer-reviewed journals. At the end of the research period, data will be retained by the researcher and will not be sold to a third party but could be shared for further research.

Right to Withdraw

You are free to refuse to participate in the research and to withdraw from this study at any time. Your decision to withdraw will bring no negative consequences — no penalty to you.

Right to ask Questions

You are free to ask questions about the research without any negative consequences to you.

Informed Consent

By clicking "Next" below you confirm that you have read the description, including the purpose of the study, the procedure to be used, the information concerning the potential risks and side effects, confidentiality issues, as well as the option to withdraw from the study at any time. Furthermore, you confirm that each of these items has been explained to you by the investigator(s) if required, and the investigator(s) answered all your questions regarding the study, and you believe you hav understood what is involved.

By clicking "Next" below you indicate that you freely agree to participate in this study.

I DO NOT ACCEPT | I HAVE READ AND I ACCEPT

Session presentation

Welcome to this session.

You are going to taste 4 wines while being video recorded and answer some questions.

Each tasting phase will be recorded on video.

You will only be recorded during the phases in which your face will be visible on the screen.

The whole activity takes just 20 minutes, and your responses are completely anonymous.

A pop up should ask you if Timesens can access your webcam, please accept.

Please also read carefully the instructions on each screen.

If you have any question about the survey, please email us: magalie.dubois@u-bordeaux.fr

Thank you for taking the time to participate in this research, we really appreciate your input!

NEXT

Berria
2018
Bhilar
Rioja
Denominación de Origen Calificada
Conditionné par Vinovae à Lyon France pour l'Université de Bordeaux
Produit d'Espagne
Lot :1/45/1170
Contient des sulfites
20 ml
13,5% vol

Château Féret-Lambert
2018
Bordeaux Supérieur
Appellation d'Origine Contrôlée
Conditionné par Vinovae à Lyon France pour l'Université de Bordeaux
Produit de France
Lot :L08-21/1/45/1173
Contient des sulfites
20 ml
14,5% vol

Viñas Jóvenes
2018
Miguel Merino
Rioja
Denominación de Origen Calificada
Conditionné par Vinovae à Lyon France pour l'Université de Bordeaux
Produit d'Espagne
Lot :1/45/1172
Contient des sulfites
20 ml
14% vol

L de la Louvière
2018
Vignobles André Lurton
Pessac-Léognan
Appellation d'Origine Contrôlée
Conditionné par Vinovae à Lyon France pour l'Université de Bordeaux
Produit de France
Lot :1/45/1171
Contient des sulfites
20 ml
13,5% vol

How much do you think you will like these wines?

	Expect to dislike extremely	Expect to dislike moderately	Expect to dislike slightly	Expect to neither like nor dislike	Expect to like slightly	Expect to like moderately	Expect to like extremely
Viñas Jovenes	☐	☐	☐	☐	☐	☐	☐
Château Féret Lambert	☐	☐	☐	☐	☐	☐	☐
L de la Louvière	☐	☐	☐	☐	☐	☐	☐
Berria	☐	☐	☐	☐	☐	☐	☐

NEXT

Question [EL1]

Preparation

Please bring the set of 4 wine samples.

Prepare a glass of water and an empty wine glass.

When you are ready, click "next".

NEXT

Instructions for webcam calibration

Please make the conditions optimum for the video recording:

- Face the webcam (ideally at the height of the eyes)
- Make your face and forefront visible
- Adapt the lighting to make it homogeneous and ideally coming from behind the webcam (if you can, just place your computer in front of a window to capture natural light)
- Your face must occupy 25% to 30% of the screen
- Avoid white clothes, direct lighting to the face, dark environments, hairstyles covering too much of the face, touching the face or resting chins on your hands
- Please turn off you phone for the duration of the study to ensure proper testing conditions and limit distractions

NEXT

Checking

The following conditions are verified:

- ☐ I avoided: wearing white clothes, direct lighting of my face, a dark environment
- ☐ I turned off my phone for the duration of the study
- ☐ My face occupies 25% to 30% of the screen
- ☐ The lighting is homogeneous
- ☐ My face and forehead are visible
- ☐ I face the webcam

NEXT

Calibration

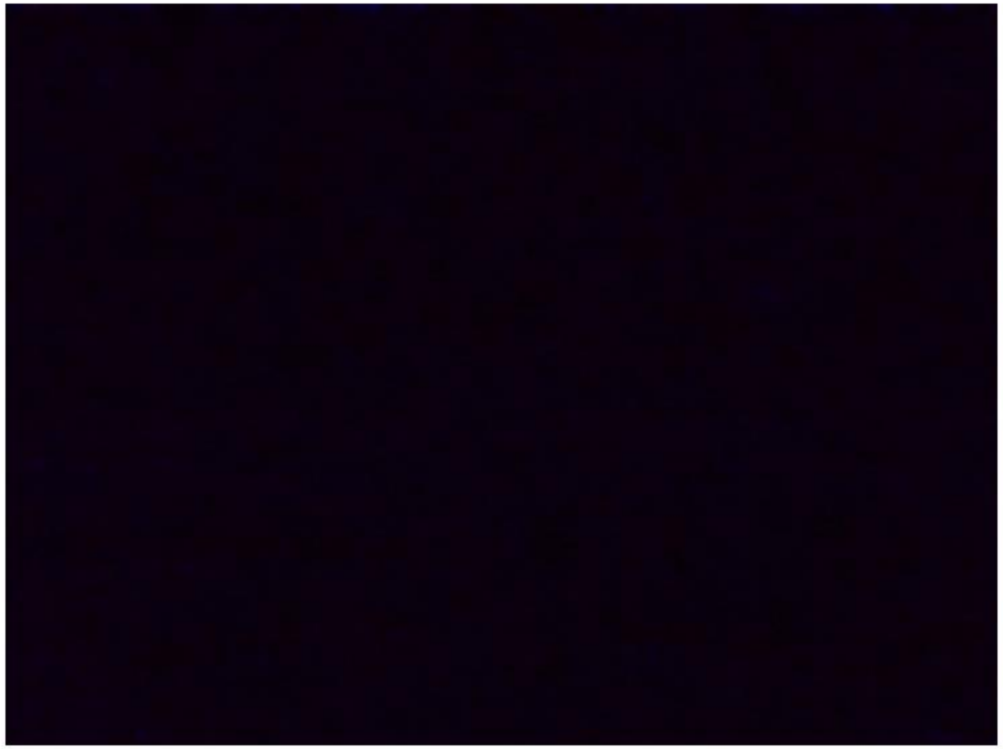

NEXT

Test - Water tasting

In order to test the quality of your video recording, please pour a bit of water in you wine glass.

Do not drink right now.

First look at the water.

Next swirl and sniff the water.

Then, when you are ready to taste, face the camera, take a small mouthfull, and at the same time, press start button. The video recording will be launched.

NEXT

Test - Water tasting

Consider the taste during the video recording

You are being video recorded during 10 seconds

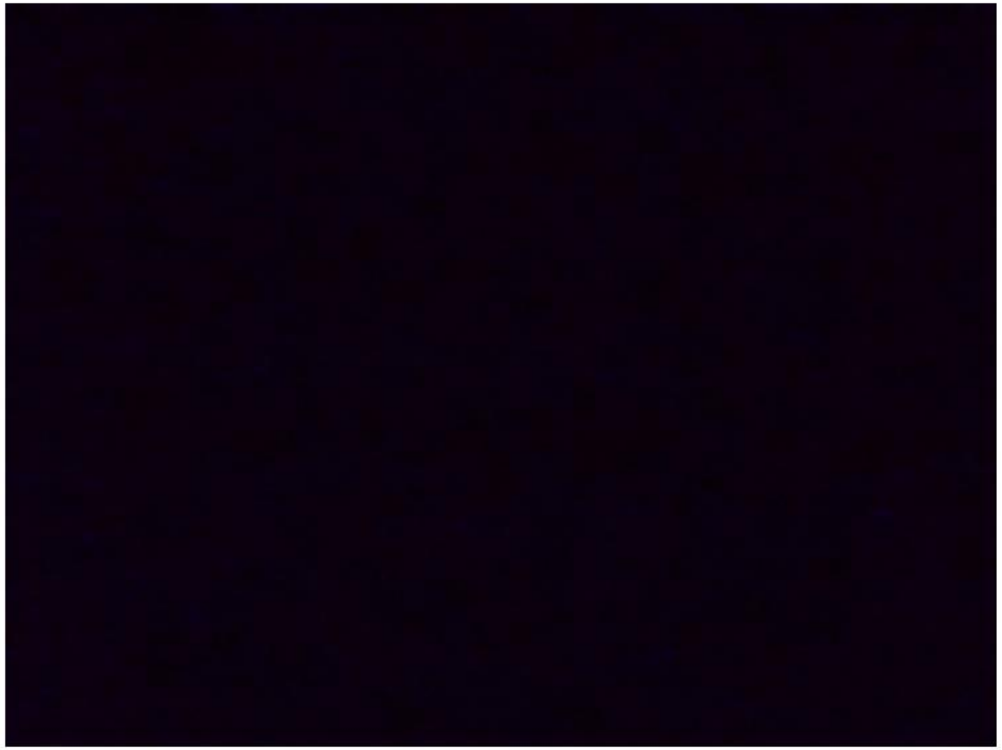

Wine tasting

First, empty your wine glass.

Pour wine **Viñas Jovenes** in you wine glass.

Do not drink right now.

First look at the wine.

Next swirl and sniff the wine.

Then, when you are ready, face the camera, take a small mouthfull, and at the same time, press the start button. The video recording will be launched.

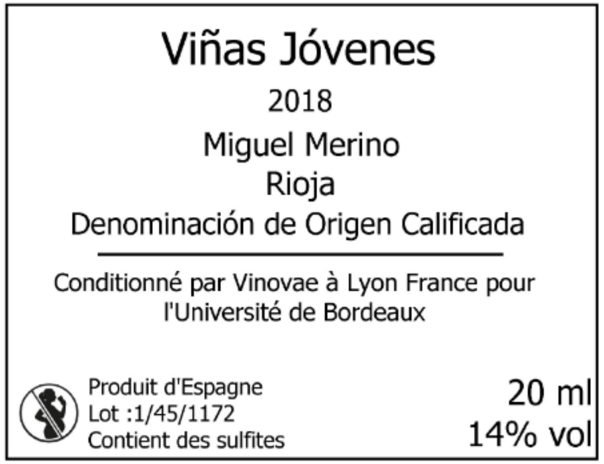

START

Group "No information"

Wine tasting

First, empty your wine glass.

Pour wine **Viñas Jovenes** lin you wine glass.

Do not drink right now.

First look at the wine.

Next swirl and sniff the wine.

Then, when you are ready, face the camera, take a small mouthfull, and at the same time, press the start button. The video recording will be launched.

Viñas Jóvenes
2018
Miguel Merino
Rioja
Denominación de Origen Calificada

Conditionné par Vinovae à Lyon France pour l'Université de Bordeaux

Produit d'Espagne
Lot :1/45/1172
Contient des sulfites

20 ml
14% vol

This wine was scored **90/100**

by "The Wine Advocate" - Robert Parker

START

Group "Expert rating information"

université de BORDEAUX

Wine tasting

First, empty your wine glass.

Pour wine **Viñas Jovenes** lin you wine glass.

Do not drink right now.

First look at the wine.

Next swirl and sniff the wine.

Then, when you are ready, face the camera, take a small mouthfull, and at the same time, press the start button. The video recording will be launched.

Viñas Jóvenes
2018
Miguel Merino
Rioja
Denominación de Origen Calificada

Conditionné par Vinovae à Lyon France pour l'Université de Bordeaux

Produit d'Espagne
Lot :1/45/1172
Contient des sulfites

20 ml
14% vol

This wine was scored **4.10/5**

by consumers - Website Vivino

START

Group "Consumer rating information"

Wine tasting

Consider the taste during the video recording

You are being video recorded during 10 seconds

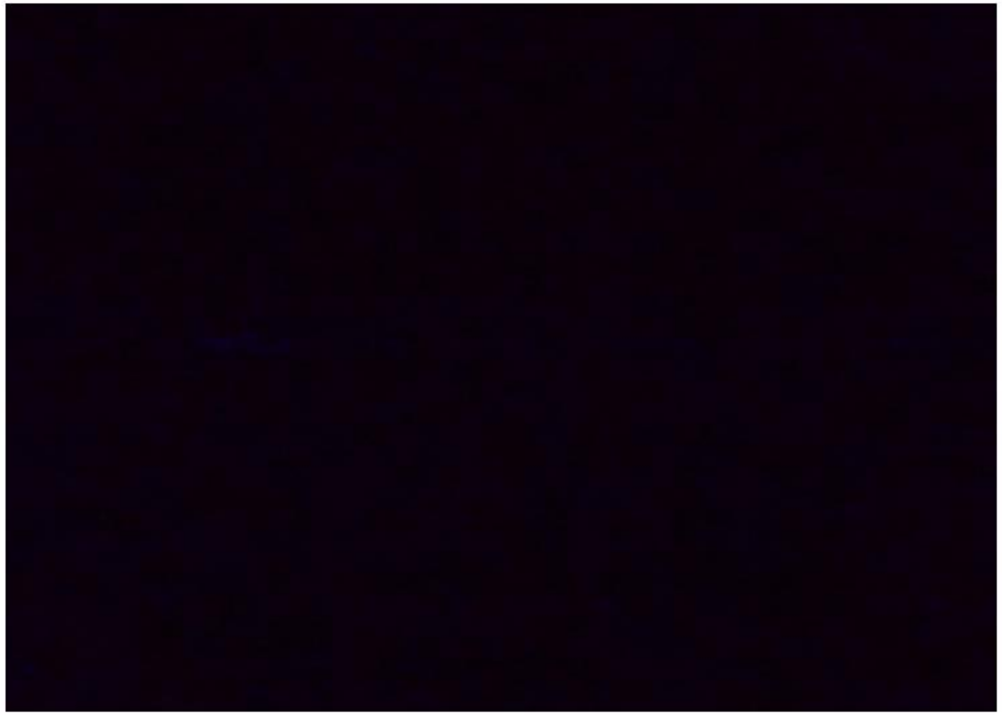

Hedonic related Tasting question

How much did you like this wine?

dislike extremely	dislike moderately	dislike slightly	neither like nor dislike	like slightly	like moderately	like extremely
☐	☐	☐	☐	☐	☐	☐

NEXT

Question SL1

Wine description

On the next screen, we will ask you to describe the sensations you perceived during the tasting (tastes, aromas) in chronological order.

Use your own words to answer. You can use the same word at different times. If you haven't perceived anything for a period, enter "nothing".

Fictitious example on chocolate:

At first I perceived this chocolate to

crunchy

Then after a few moments I perceived it to

melty, cocoa, sweet

And at the end of the tasting I perceived it to

cocoa, bitter

NEXT

Wine description

Describe the sensations you perceived during the tasting (tastes, aromas) in chronological order. Use your own words to answer. You can use the same word at different times. If you haven't perceived anything for a period, enter "nothing".

At first I perceived this wine to be

Then after a few moments I perceived it to be

And at the end of the tasting I perceived it to be

NEXT

Questions AEF1, AEF2, AEF3

This wine profile is familiar to me

Strongly disagree	Disagree	Neither agree nor disagree	Agree	Strongly agree
☐	☐	☐	☐	☐

NEXT

Question F1

I think this wine is high quality

Strongly disagree	Disagree	Neither agree nor disagree	Agree	Strongly agree
☐	☐	☐	☐	☐

Most people would like this wine

Strongly disagree	Disagree	Neither agree nor disagree	Agree	Strongly agree
☐	☐	☐	☐	☐

NEXT

Questions QE1, QE2

Auction

You have been recruited because you have declared that you buy and consume red wine.

We are interested in the determinants of purchases, so we are going to put you in a real purchase condition.

To do this, we will ask you to propose a price for each of the red wines presented to you, after having tasted them.

The price you quote should be the maximum price you are willing to pay for each bottle.

It is possible that you do not like the products proposed at all. In this case, you can indicate 0 to show that you do not wish to buy the product.

Only one of the 4 wines will be drawn at the end of the survey.

The price of this wine will also be drawn.

- If the price drawn is higher than the price you indicated, you will lose the auction and will not receive the wine.
- If the price drawn is lower than the price you indicated, you win the auction and will receive the bottle in the next few days.

To continue click on "next".

NEXT

What is the maximum price (in €uros) you are willing to pay for a 75cl bottle of the wine you just tasted? (numbers only, separated by '.')

NEXT

Question BDM1

Pour a bit of water into your glass to rinse it, then empty the glass before going to the next step

NEXT

Screens 10 to 19
repeated for each wine sample
according to a balanced design

Quality ranking

université de BORDEAUX

Rank the 4 wines by clicking on "click to choose a wine" and then affecting each wine a rank. #1=most qualitative wine, #4=less qualitative wine

#1 Click to select a wine

#2 Click to select a wine

#3 Click to select a wine

#4 Click to select a wine

NEXT

Question RQ1

Wine interests me a lot

Strongly disagree	Disagree	Neither agree nor disagree	Agree	Strongly agree
☐	☐	☐	☐	☐

I often discuss wine with other people

Strongly disagree	Disagree	Neither agree nor disagree	Agree	Strongly agree
☐	☐	☐	☐	☐

It gives me pleasure to shop for wine

Strongly disagree	Disagree	Neither agree nor disagree	Agree	Strongly agree
☐	☐	☐	☐	☐

NEXT

Questions PCI1, PCI2, PCI3

I feel confident in my ability to choose wine

Strongly disagree ☐ | Disagree ☐ | Neither agree nor disagree ☐ | Agree ☐ | Strongly agree ☐

I know more about wine than many other people

Strongly disagree ☐ | Disagree ☐ | Neither agree nor disagree ☐ | Agree ☐ | Strongly agree ☐

I would describe myself as being very knowledgeable about wine

Strongly disagree ☐ | Disagree ☐ | Neither agree nor disagree ☐ | Agree ☐ | Strongly agree ☐

Did you already attend a wine education course?

Please select an option

NEXT

Questions SWK1, SWK2, SWK3, SWK4

I use wine apps to help me decide which wine to buy

- ☐ never
- ☐ once in a while
- ☐ often
- ☐ always

I use wine professional experts ratings (wine reviews, point scores, medals and awards) to help me decide which wine to buy

- ☐ never
- ☐ once in a while
- ☐ often
- ☐ always

NEXT

Questions VB1, VB2

I often seek advice from other people before purchasing a wine

Strongly disagree	Disagree	Neither agree nor disagree	Agree	Strongly agree
☐	☐	☐	☐	☐

NEXT

Question VB3

Whose advice do you trust most when selecting a wine? (you can select more than one option)

- ☐ Friends
- ☐ Family members
- ☐ Colleague
- ☐ Sommelier
- ☐ Professional Wine Expert
- ☐ Wine blogger or influencer
- ☐ Wine Guide or Magazine
- ☐ Only my own

NEXT

Question VB4

Where do you buy your wine? (you can select more than one option)

- ☐ I don't buy wine
- ☐ Supermarket
- ☐ Wine store
- ☐ Online
- ☐ Directly to the winemaker
- ☐ Restaurants & Bars
- ☐ Other

NEXT

Question PP1

For a 75cl bottle of red wine you spend on average - for informal drinking

Click to select an option ⌄

For a 75cl bottle of red wine you spend on average - for formal occasion or for a gift

Please select an option ⌄

How much does your household spend on wine monthly?

Please select an option ⌄

NEXT

Questions PP2, PP3, PP4

How often do you consume wine at home?

Please select an option

How often do you consume wine outside from home (restaurant, bar, club…)?

Please select an option

If you read this sentence correctly, please answer "Strongly disagree"

Strongly disagree	Disagree	Neither agree nor disagree	Agree	Strongly agree
☐	☐	☐	☐	☐

NEXT

Questions CP1, CP2, AC1

Drawed product: Château Féret Lambert

Your reserve price: 0

Random price: 17.10

Result : Purchase not feasible!

NEXT

Debriefing

The purpose of this study is to investigate the role of information provision about wine quality on participants' attitudes towards wine and willingness to pay. The information presented to you is real data. There are several versions of this presentation (including the one you have just read).

We are looking to see if participants give different answers depending on the source of the information presented.

We would like to remind you that no personal data that could identify you is recorded and that the data available will be used for scientific research purposes only.

In order for this research to be carried out in the best possible conditions, and for the results to remain comparable between participants over time, we ask you to keep the objectives of this study confidential.

Thank you for participating in this study. If you have any questions about this research, or if you would like further information, please send an email to: magalie.dubois@u-bordeaux.fr

NEXT

The questionnaire is complete.

Thank you for your participation.
